# Heart rate variability is associated with cerebral small vessel disease in patients with diabetes

**DOI:** 10.3389/fneur.2022.989064

**Published:** 2022-11-10

**Authors:** Qianwen Qiu, Wenhui Song, Xirui Zhou, Zhiyuan Yu, Minghuan Wang, Huang Hao, Dengji Pan, Xiang Luo

**Affiliations:** ^1^Department of Neurology, Tongji Hospital, Tongji Medical College, Huazhong University of Science and Technology, Wuhan, China; ^2^Department of Neurology, The Central Hospital of Wuhan, Tongji Medical College, Huazhong University of Science and Technology, Wuhan, China

**Keywords:** cerebral small vessel disease, heart rate variability, autonomic nervous system, diabetes, white matter hyperintensity

## Abstract

**Objective:**

Low heart rate variability (HRV), an indicator of autonomic nervous system dysfunction, has been associated with increased all-cause and cardiovascular mortality and incident stroke. However, the relationship between HRV and cerebral small vessel disease (CSVD) showed contradictory results. We aimed to examine the relationship of HRV and total burden of CSVD and each of the magnetic resonance imaging (MRI) markers of CSVD.

**Methods:**

We recruited 435 patients who attended our hospital for physical examination between June 2020 and August 2021. All underwent 24-h Holter monitoring and MRI scan. The standard deviation of normal-to-normal intervals (SDNN) was selected as the method for HRV assessment. The presence of severe white matter hyperintensity, lacunes, and >10 enlarged basal ganglia perivascular spaces, and cerebral microbleeds were added for estimating the CSVD score (0–4). Multivariate logistic analyses was performed to assess whether HRV was independently associated with the burden of CSVD and each of the MRI markers of CSVD, with and without stratification by prevalent diabetes.

**Results:**

This study included 435 subjects with a mean age of 64.0 (57.0–70.0) years; 49.4% of the patients were male, and 122 (28.0%) had a history of diabetes. In multivariate analyses, lower SDNN was independently associated with total burden of CSVD and the presence of enlarged perivascular spaces in all subjects. According to diabetes stratification, lower SDNN was independently associated with total burden of CSVD and each MRI markers of CSVD separately only in the diabetic group.

**Conclusions:**

Lower HRV was associated with total burden of CSVD and each MRI markers of CSVD separately among diabetic patients, but not among non-diabetic patients.

## Introduction

White matter hyperintensity (WMH), lacunes, enlarged perivascular spaces (EPVS), and cerebral microbleeds (CMBs) have all been identified as markers of cerebral small vessel disease (CSVD) of presumed vascular origin, which are commonly detected on brain magnetic resonance imaging (MRI) in the elderly (1). Previous studies have reported that CSVD is associated with increased risk of cognitive impairment and physical disability (2). Despite extensive studies, there are still controversies and lack of knowledge on pathogenic mechanisms underlying the development of CSVD (3). Numerous studies have suggested traditional risk factors for CSVD–most prominently age, sex, hypertension, diabetes hypercholesterolaemia, and smoking (4). Recent studies have focused on questing for underappreciated risk factors of CSVD development and progression to facilitate the development of prevention and treatment strategies for CSVD. Autonomic dysfunction is an increasingly recognized non-traditional risk factor of CSVD. Prior studies have suggested that indicators of autonomic dysfunction are significantly associated with the risk of neurological progression in CVSD patients, such as cognitive decline (5, 6). A community-based longitudinal study found that higher 24-h systolic coefficient of variation (CV), diastolic weighted standard deviation (SD), and diastolic CV were significant predictors of CSVD progression. Another prospective case-control study showed that 24-h mean systolic blood pressure (SBP) and nocturnal mean SBP and diastolic blood pressure (DBP) were significantly correlated with CSVD (5, 6).

Heart rate variability (HRV) is a commonly used indicator of autonomic nervous system (ANS) function and has been widely applied in multiple risk stratification models in patients with cardiovascular diseases (7). More recently, lower HRV has been related to higher risk of cerebrovascular diseases, such as incident stroke and secondary ischemic events (8, 9). The primary mechanisms proposed include the effects of ANS on cerebral circulation autoregulation, blood pressure and essential hypertension (10, 11). Previous studies have reported conflicting results regarding the relationship between HRV and CSVD. A community-based study showed that higher HRV is associated with WMH, thereby suggesting that ANS dysfunction is related to CSVD development (12). However, another study reported that low nighttime HRV was associated with a greater CSVD burden (13). These studies mostly focused on WMH and lacunes, did not investigate the results of CMBs and EPVS (12, 13). There was only one study that provided an overall measure for the total burden of CSVD (13). From a clinical perspective, it is important to explore these issues, as HRV may represent a target for CSVD prevention through lifestyle changes and medication or that improve or preserve ANS function.

Moreover, the association between HRV and cardiovascular and cerebrovascular events and mortality has been shown to be stronger in people with diabetes (7, 14). Identifying factors related to the poor cerebrovascular prognosis of diabetic patients may lead to more effective screening and earlier institution of primary prevention programs to slow down the development and progress of cerebrovascular disease in diabetic patients. Therefore, the purpose of this study was to investigate the relationship of HRV and total burden of CSVD and each of the MRI markers of CSVD by stratification of diabetes.

## Methods

This was a cross-sectional study conducted in the Department of Neurology, Tongji Hospital, Tongji Medical College, Huazhong University of Science and Technology, China, between June 2020 and August 2021. Patients attended to the neurological clinic and were electively admitted to hospital for physical examination because of headache, dizziness or with the fear of stroke due to the presence of risk factors. Patients with CSVD were recommended to undergo 24-h Holter preferentially. Patients were included if they underwent 24-h Holter monitoring and MRI scan. Patients were excluded if they were aged < 50 years or had history of heart disease (e.g., ischemic heart disease or cardiac diseases such as heart failure, valvular heart disease, congenital heart disease, or arrhythmias), stroke, large-vessel cerebrovascular diseases (i.e., extracranial or intracranial large artery stenosis > 50%), cancer, or other significant or life-threatening diseases, such as severe nephrosis or liver diseases.

### Collection of clinical characteristics

**Figure 1:**
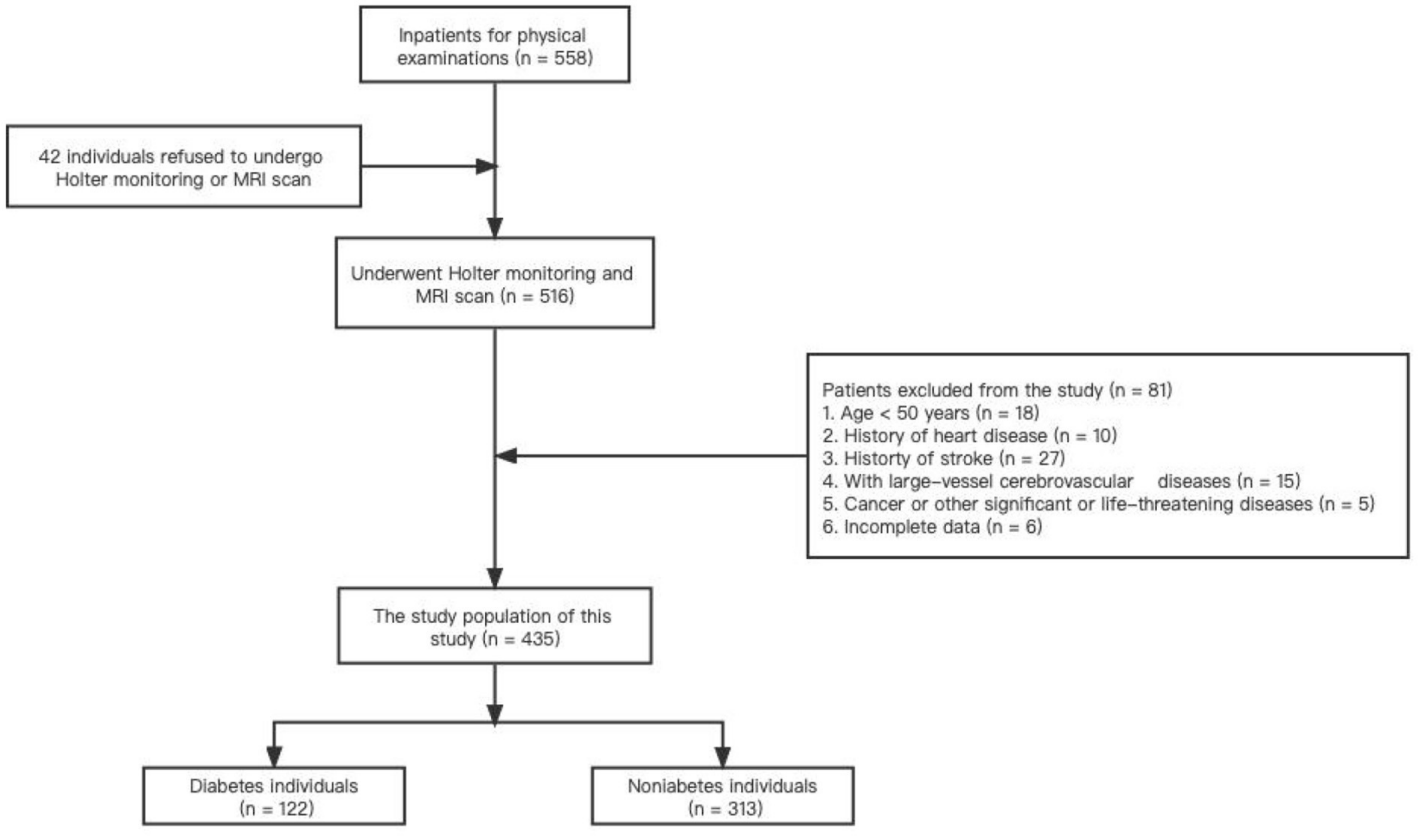
Flowchart of selection of participants.

Basic information of all patients were recorded, including age, sex, medical history, smoking status, alcohol intake, and levels of fasting blood glucose, glycosylated hemoglobin A1c (HbA1c), triglyceride (TG), total cholesterol (TC), high-density lipoprotein (HDL) cholesterol, and low-density lipoprotein (LDL) cholesterol. Diabetes mellitus was defined as HbA1c ≥ 6.5%, fasting blood glucose readings of >7.0 mmol/L, or a previous diagnosis of type I or type II diabetes. Hypertension was defined as having DBP ≥ 90 mm Hg, SBP ≥ 140 mm Hg, or taking antihypertensive medication. Hyperlipidemia was defined by TG ≥ 1.7 mmol/L, TC ≥ 5.2 mmol/L, a previous diagnosis of hyperlipidemia, or current use of lipid lowering drugs. The selection criteria for the patients are presented in Figure 1.

### Heart rate variability

Electrocardiographic recordings were acquired using 24-h Holter monitoring (DMS 300-4, Holter Reader, Producer DMS, Nevada, USA). The parameters were automatically recorded by the Holter and analyzed by Cardioscan 12, HRV Package system. Time domain measurement, defined as the SD of normal-to-normal (NN) intervals (SDNN), which characterizes overall HRV, was selected as the method for HRV assessment.

### MRI protocol and assessments

Brain MRI was performed using a 3.0 T scanner (Signa, GE Healthcare of America, Milwaukee, WI, USA). The scan protocol included diffusion-weighted, T1-weighted, T2-weighted, and fluid-attenuated inversion recovery (FLAIR) sequences. The parameters of conventional MRI sequences are shown in Supplementary Table S1. The scans were rated by two radiologists trained in MRI assessments and blinded to the clinical data. Any disagreements were resolved through discussion with a third radiologist. Interrater reliability tests were performed in 50 subjects for each CSVD marker assessment, and the κ = 0.763–0.895 indicating good reliability.

WMH, lacunes, EPVS, and CMBs were defined as described previously (15). The Fazekas scale (0–6) was used to rate the periventricular and deep WMH on the FLAIR sequence (16). Lacunes were distinguished from EPVS by their size (>3 mm), spheroid shape, location, and surrounding hyperintensity on FLAIR and T2-weighted imaging (17). EPVS were only counted in basal ganglia level because in this level they are known to be closely associated with CSVD (18).

On the CSVD burden scale (0–4), one point was awarded for each of the following neuroimaging signature: severe WMH (periventricular WMH Fazekas score 3 or deep WMH Fazekas score 2 or 3), presence of one or more lacunes, >10 basal ganglia EPVS on one side of the brain and presence of one or more CMBs (18).

### Statistical analysis

Statistical analysis were performed using SPSS software version 22.0. For non-normally distributed variables, median value and quartiles (Q1–Q3) are presented. Categorical variables are presented as numbers and percentages. The normality of data was checked using one-sample Kolmogorov-Smirnov test. Differences between groups were determined using the Mann-Whitney *U-*test, the Kruskal-Wallis test, or the χ^2^-test where appropriate. The generalized linear model (GLM) was used to test the correlation of HRV with CSVD score. Thereafter, we performed ordinal logistic regression analyses and binary logistic regression analyses (forward LR), respectively, to determine whether the HRV was independently associated with the total burden of CSVD and each CSVD MRI markers. Demographic and confounding factors were adjusted in the model as follows: the model 1 was adjusted for age and sex; and the model 2 was adjusted for age, sex, current smoking, alcohol use, history of hypertension, using of ß-receptor blocker, using of lipid lowering drugs, SBP, HbA1c, LDL, and HR levels. There was no multicollinearity among variables in adjusted models. *P*-value < 0.05 was considered statistically significant.

## Results

### Demographic and clinical characteristics

Among 558 patients admitted for physical examination, 435 fulfilled the selection criteria (Figure 1). Table 1 presents the demographic and clinical characteristics of the study population. The average age of the patients was 64.0 (57.0–70.0) years, 49.4% of the patients were male, and 309 (71.0%), 122 (28.0 %), and 160 (36.8%) had a history of hypertension, diabetes, and hyperlipidemia, respectively. Severe WMH, lacunes, EPVS, and CMBs were present in 288 (66.2%), 256 (58.9%), 273 (62.8%), and 133 (30.2%) patients, respectively. The overall median of SDNN for was 112.0 (94.0–132.0) ms. All included subjects were divided into two groups based on diabetic status. Diabetic group accounts for 28.0% of all involved subjects. Diabetic group presented with higher frequencies of male, smoking, alcohol use, hypertension, using of lipid lowering drugs, and presence of CMBs than the non-diabetic group. Diabetic group had higher levels of SBP, glucose, HbA1c, TG, and CSVD score, but lower levels of HDL and SDNN. There was no statistically significant difference in other characteristics. Each group was divided into five subgroups according to the total CSVD burden and their characteristics are presented in Supplementary Tables S2–S4, respectively. Age, the prevalence of hypertension, and the presence of each CSVD MRI marker increased with increasing CSVD score in three groups. In all subjects and the non-diabetic group, there were statistical differences in frequencies of using of ß-receptor blocker among different CSVD burden subgroups.

**Table 1:** Characteristics of the diabetic patients and according to total burden of CSVD.

	**Total** ***n* = 435**	**Diabetic patients** ***n* = 122**	**Non-diabetic patients** ***n* = 313**	***P*-value**
Age, years, median (IQR)	64.0 (57.0–70.0)	65.0 (57.8–71.3)	63.0 (57.0–69.0)	0.207
Male sex, *n* (%)	215 (49.4)	70 (57.4)	145 (46.3)	0.038
Smoking, *n* (%)	157 (36.1)	58 (47.5)	99 (31.6)	0.002
Alcohol use, *n* (%)	114 (26.2)	42 (34.4)	72 (23.0)	0.015
Hypertension, *n* (%)	309 (71.0)	103 (84.4)	206 (65.8)	<0.001
Hyperlipidemia, *n* (%)	160 (36.8)	50 (41.0)	110 (35.1)	0.257
Using of ß-receptor blocker, *n* (%)	60 (13.8)	14 (11.5)	46 (14.6)	0.381
Using of lipid lowering drugs, *n* (%)	103 (23.7)	39 (32.0)	64 (20.4)	0.011
SBP, mm Hg, median (IQR)	133.0 (125.0–143.0)	140.0 (128.0–149.0)	132.0 (124.0–141.0)	0.001
DBP, mm Hg, median (IQR)	79.0 (73.0–85.0)	78.0 (73.0–83.0)	79.0 (72.0–85.0)	0.782
Glucose, mmol/L, median (IQR)	5.8 (5.5–6.2)	6.8 (6.3–7.6)	5.7 (5.5–5.9)	<0.001
HbA1c level, %, median (IQR)	5.2 (4.8–5.7)	6.2 (5.3–7.5)	5.0 (4.7–5.4)	<0.001
TC, mmol/L, median (IQR)	3.8 (3.1–4.5)	3.7 (2.9–4.4)	3.9 (3.2–4.6)	0.063
TG, mmol/L, median (IQR)	1.2 (0.9–1.7)	1.3 (1.0–1.9)	1.1 (0.8–1.6)	0.004
HDL, mmol/L, median (IQR)	1.1 (0.9–1.3)	1.0 (0.9–1.2)	1.1 (0.9–1.3)	0.001
LDL, mmol/L, median (IQR)	2.3 (1.7–2.3)	2.2 (1.5–2.8)	2.4 (1.8–3.0)	0.070
**CSVD markers**
Presence of severe WMH, *n* (%)	288 (66.2)	78 (63.9)	210 (67.1)	0.532
Presence of Lacunes, *n* (%)	256 (58.9)	79 (64.8)	177 (56.5)	0.118
Presence of EPVS, *n* (%)	273 (62.8)	90 (73.8)	183 (58.5)	0.003
Presence of CMBs, *n* (%)	133 (30.2)	45 (36.9)	88 (28.1)	0.075
CSVD score, median (IQR)	2 (1–3)	3 (2–3)	2 (1–3)	0.021
HR levels, times/min, median (IQR)	72.0 (62.0–82.0)	76.0 (65.0–87)	69.0 (62.0–76)	0.002
SDNN, ms, median (IQR)	112.0 (94.0–131.0)	107.5 (88.8–120.3)	115.0 (98.0–134.0)	<0.001

SBP, systolic blood pressure; DBP, diastolic blood pressure; TC, total cholesterol; TG, triglyceride; HDL, high-density lipoprotein; LDL, low-density lipoprotein; HAb1c, hemoglobin A1c; WMH, white matter hyperintensity; EPVS, enlarged perivascular spaces; CMBs, cerebral microbleeds; SDNN, SD of normal-to-normal intervals.

### Association between HRV and total burden of CSVD

**Figure 2:**
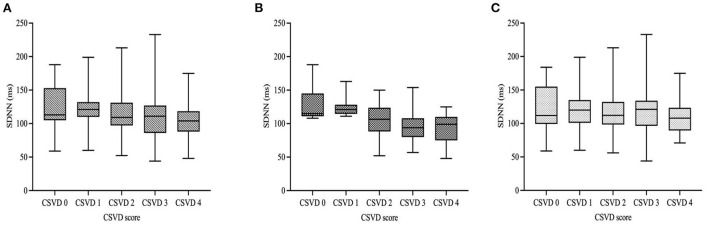
HRV values of the patients according to diabetic status and total burden of CSVD. In all subjects and the diabetic group, there was statistical difference in SDNN among the five subgroups but no difference was found in the non-diabetic group [**(A–C)**; *p* < 0.001; *p* < 0.001; *p* = 0.218, respectively]. GLM indicated that SDNN was negatively correlated with the total CSVD burden in all subjects with different diabetic status. [**(A–C)**; β = −0.198, 95% CI = −0.264 to −0.092, *p* = 0.001; β = −0.216, 95% CI = −0.351 to −0.045, *p* = 0.001; β = −0.122, 95% CI = −0.236 to −0.058, *p* = 0.036, respectively]. Boxplot elements: center line represents the median, box bounds represent the 25th and 75th percentile, and whiskers represent minimum and maximum, respectively. SDNN, SD of normal-to-normal intervals.

Figure 2 showed HRV values of the patients with different total burden of CSVD according to the stratification of diabetes. We found that there was statistical difference in SDNN among the five subgroups of all subjects, which decreased as the burden of CSVD increased. GLM also indicated that SDNN was negatively correlated with the total CSVD burden in all subjects (β = −0.198, 95% CI = −0.264 to −0.092, *p* = 0.001, Figure 2A). According to diabetes stratification, SDNN was significantly different among the five subgroups of the diabetic group, but not in the non-diabetic group. GLM indicated that SDNN was negatively correlated with the total burden of CSVD in the two groups (Figures 2B,C; β = −0.216, 95% CI = −0.351 to −0.045, *p* = 0.001; β = −0.122, 95% CI = −0.236 to −0.058, *p* = 0.036, respectively).

Table 2 presented the results from the mulitivariate ordinal logistic regression models. After adjustment for age and sex, SDNN was significantly associated with total CSVD burden [adjusted odds ratio (aOR) = 0.983; 95% confidence interval (CI) = 0.969–0.994, *p* = 0.001] in all subjects. This association remained significant when we additionally adjusted for current smoking, alcohol use, history of hypertension, using of ß-receptor blocker, using of lipid lowering drugs, SBP, HbA1c, LDL and HR levels (aOR = 0.968; 95% CI = 0.945–0.995, *p* = 0.006). According to diabetes stratification, SDNN was independently associated with total CSVD burden (aOR = 0.955; 95% CI = 0.948–0.982, *p* = 0.003) in diabetic group after adjusting for confounding factors included in the model 2. There was no association between SDNN with total CSVD burden in non-diabetic group.

**Table 2:** HRV in relation to the total burden of CSVD according to diabetic status stratification.

	**Mulitivariate analyses**			
**Variables**	**Model 1**		**Model 2**	
	**aOR** ** (95% CI)**	***P*-value**	**aOR** ** (95% CI)**	***P*-value**
**All subjects**
CSVD score	0.983 (0.969–0.994)	0.001	0.968 (0.945–0.995)	0.002
**Diabetic patients**
CSVD score	0.959 (0.947–0.980)	<0.001	0.955 (0.948–0.982)	0.001
**Non-diabetic patients**
CSVD score	0.990 (0.982–1.002)	0.487	0.967 (0.965–0.992)	0.465

Model 1: adjusted for age and sex.

Model 2: adjusted for age, sex, current smoking, alcohol use, history of hypertension, using of ß-receptor blocker, using of lipid lowering drugs, SBP, HbA1c, LDL, and HR levels.

WMH, white matter hyperintensity; EPVS, enlarged perivascular spaces; CMBs, cerebral microbleeds; SBP, systolic blood pressure; HAb1c, hemoglobin A1c; LDL, low-density lipoprotein.

### Association between HRV and different MRI markers of CSVD

Binary logistic regression models were used to assess association between SDNN and different MRI markers of CSVD. As shown in Table 3, for all subjects, univariate logistic analyses showed that SDNN was related to each MRI markers of CSVD separately. Mulitivariate logistic analyses demonstrated that SDNN was only independently related to the presence of EPVS (aOR = 0.977; 95% CI = 0.950–0.989, *p* = 0.010). According to diabetes stratification, univariate logistic analyses indicated that SDNN was associated with each MRI markers of CSVD separately in diabetic group. But in the non-diabetic group, SDNN was only associated with the presence of EPVS. Subsequently, in the mulitivariate logistic regression models, SDNN was significantly associated with each MRI markers of CSVD separately in diabetic group after adjustment for age and sex. After additional adjustment for vascular risk factors, SDNN was still related to each MRI markers of CSVD separately in diabetic group (severe WMH: aOR = 0.955; 95% CI = 0.944–0.993, *p* = 0.015; lacunes: aOR = 0.966; 95% CI = 0.952–0.996, *p* = 0.021 EPVS: aOR = 0.962; 95% CI = 0.946–0.986, *p* = 0.011; CMBs: aOR = 0.967; 95% CI = 0.950–0.987, *p* = 0.018). However, we found no independent association between SDNN and any MRI markers of CSVD in non-diabetic group.

**Table 3:** HRV in relation to different CSVD markers according to diabetic status stratification.

	**Univariate analyses**		**Mulitivariate analyses**			
**Variables**			**Model 1**		**Model 2**	
	**OR (95% CI)**	***P*-value**	**aOR (95% CI)**	***P*-value**	**aOR (95% CI)**	***P*-value**
**All subjects**
Severe WMH	0.991 (0.984–0.998)	0.013	0.981 (0.944–0.989)	0.173	0.942 (0.932–0.969)	0.214
Lacunes	0.992 (0.986–0.999)	0.025	0.983 (0.966–0.979)	0.189	0.953 (0.934–0.976)	0.343
EPVS	0.985 (0.978–0.992)	<0.001	0.979 (0.962–0.989)	0.003	0.977 (0.950–0.989)	0.010
CMBs	0.989 (0.982–0.997)	0.004	0.968 (0.976–0.983)	0.082	0.967 (0.961–0.982)	0.132
**Diabetic patients**
Severe WMH	0.976 (0.959–0.993)	0.005	0.988 (0.976–0.998)	0.005	0.955 (0.944–0.993)	0.015
Lacunes	0.970 (0.953–0.988)	0.001	0.965 (0.945–0.987)	0.014	0.966 (0.952–0.996)	0.021
EPVS	0.962 (0.942–0.983)	<0.001	0.976 (0.964–0.982)	0.006	0.962 (0.946–0.986)	0.013
CMBs	0.971 (0.954–0.988)	0.001	0.979 (0.958–0.987)	0.008	0.967 (0.950–0.987)	0.011
**Non-diabetic patients**
Severe WMH	0.994 (0.986–1.002)	0.124	0.973 (0.967–0.992)	0.381	0.963 (0.955–0.984)	0.435
Lacunes	0.998 (0.991–1.006)	0.608	0.975 (0.969–0.995)	0.645	0.962 (0.946–0.981)	0.638
EPVS	0.990 (0.983–0.998)	0.012	0.968 (0.953–0.980)	0.176	0.964 (0.956–0.987)	0.425
CMBs	0.995 (0.987–1.004)	0.270	0.970 (0.978–0.999)	0.462	0.963 (0.957–0.989)	0.466

Model 1: adjusted for age and sex.

Model 2: adjusted for age, sex, current smoking, alcohol use, history of hypertension, using of ß-receptor blocker, using of lipid lowering drugs, SBP, HbA1c, LDL, and HR levels.

WMH, white matter hyperintensity; EPVS, enlarged perivascular spaces; CMBs, cerebral microbleeds; SBP, systolic blood pressure; HAb1c, hemoglobin A1c; LDL, low-density lipoprotein.

## Discussion

In this cross-sectional study, we investigated the relationship of HRV and total burden of CSVD and each of the MRI markers of CSVD in inpatients who underwent physical examinations in our hospital. We found that after controlling for several confounders, lower HRV was independently associated with total burden of CSVD and the presence of EPVS in all subjects. According to the stratification by diabetes mellitus status, we found that lower HRV was independently associated with an increasing total burden of CSVD and each MRI markers of CSVD separately in diabetic group, but not in non-diabetic group.

HRV quantifies the R-R interval fluctuations during the sinus rhythm, which reflects the dynamic relationship between the sympathetic and parasympathetic nervous systems (19). A lower HRV is associated with increased parasympathetic (or decreased sympathetic) activity, occurrence of multiple cardiovascular and cerebrovascular events, and increased all-cause mortality, especially among diabetic patients (7, 14). However, the results of previous studies regarding the associations of HRV and CSVD are conflicting. Consistent with our findings, a community-dwelling study showed that low HRV was related to a greater CSVD burden (13). A prospective study reported that increased HRV was associated with CSVD progression (WMH or lacunes) (12), whereas some others failed to identify an association between HRV and CSVD (20). The inconsistency between these results may be due to the differences in experimental designs in these studies. In studies suggesting a positive correlation between high HRV and risk of CSVD progression, ambulatory blood pressure monitoring was used to estimate HRV, which may be affected by intermittent cuff inflation (20). In addition, these studies did not provide the results of CMBs and EPVS. We expanded on the previous work by using HRV measure derived from 24-h Holter monitoring and assessing the association between HRV and total burden of CSVD and each of the MRI markers of CSVD, and by evaluating potential effect modification by diabetes status.

Diabetes increases risk of cardiovascular and cerebrovascular complications. In our study, we found that the vascular risk factors and comorbidity profiles of diabetic patients were higher than non-diabetic, which has been demonstrated in previous studies (21). Diabetic patients had a higher value of SDNN than non-diabetic patients, which is consistent with previous reports suggesting that ANS dysfunction is highly prevalent in patients with diabetes (22, 23). Moreover, our results are consistent with previous findings in The Atherosclerosis Risk in Communities (ARIC). Study in which diabetes was found to be an effect modifier (8): lower HRV was associated with total burden of CSVD and each of the MRI markers of CSVD separately among diabetic patients; no association was observed among non-diabetic patients. A plausible explanation for theses findings is that the previously reported relationship between low HRV and increased risk of CSVD progression may reflect the average findings for patients with and without diabetes (13).

From an etiological perspective, our findings suggest that ANS dysfunction contributes to the increased CSVD burden among diabetic patients. These markers are different imaging manifestations of CSVD but often coexist, indicating a potential shared pathological mechanism. We propose several possible explanations for the relationship between ANS dysfunction and CSVD. First, ANS controls the dynamic cerebral circulation, which plays a critical role in beat-to-beat cerebral blood flow regulation in humans (10, 11). ANS dysfunction causes cerebral circulatory dysregulation, especially cerebral blood flow reduction and cerebral hypoperfusion. In addition, ANS dysfunction has pro-atherogenic effects on the vascular function by promoting vasoconstriction, endothelial dysfunction, and oxidative stress (24). Thus, chronic hypoperfusion and arteriosclerosis due to ANS dysfunction may lead to the development of CSVD. Second, the association between HRV and CSVD may be mediated through hypertension. Sympathetic overactivity was strongly related to blood pressure regulation and onset and progression of hypertension (25). Increased ambulatory blood pressure variability was associated with resistant hypertension and CSVD progression (5, 26). Therefore, we retained blood pressure as covariates in our models to address any potential confounding effects of blood pressure on the relationship between HRV and CSVD. Finally, HRV is also associated with age, sex, smoking status, alcohol use, lifestyle factors, and other cerebrovascular factors, which together contribute to CSVD-related brain damage (27, 28). Although we did not find an association between low HRV and CSVD among non-diabetic patients, HRV values were lower in patients with higher burden of CSVD, irrespective of the presence or absence of diabetes. Therefore, low HRV may be an early indicator of impaired health and autonomic dysfunction should be treated by long-term glycemic control, smoking cessation, balanced diet, and exercise to reduce the CSVD risk, especially among diabetic patients.

The present study had several limitations. First, this is a single-center observational study with selection bias. Second, due to the limitations of cross-sectional analysis, we were unable to provide evidence for causality. Hence, further prospective longitudinal confirmation is required. Third, we did not account for the influences of antihypertensive agents on HRV, which may affect the relationships of HRV with CSVD. For example, ß-receptor blocker can affect ANS function by inhibiting sympathetic nerve. Our findings should be validated in prospective experiments with larger sample sizes.

## Conclusion

In conclusion, lower HRV was associated with total burden of CSVD and each of the MRI markers of CSVD separately in diabetic patients. Further longitudinal studies are warranted and additional exploration of the etiology of ANS dysfunction and CSVD in diabetic patients.

## Data availability statement

The raw data supporting the conclusions of this article will be made available by the authors, without undue reservation.

## Ethics statement

The studies involving human participants were reviewed and approved by the Tongji Hospital Ethics Committee (No. 2019-S105). Written informed consent was obtained from all patients. The patients/participants provided their written informed consent to participate in this study.

## Author contributions

QQ: drafting/revision of the manuscript for content, major role in the acquisition of data, study concept or design, and analysis or interpretation of data. WS and XZ: major role in the acquisition of data and analysis or interpretation of data. ZY: major role in the acquisition of data and study concept or design. MW, HH, DP, and XL: study concept or design. All authors contributed to the article and approved the submitted version.

## Funding

This study was supported by the National Nature Science Foundation of China (82171385 to XL) and Key Research and Development Program of Hubei Province (2020BCA070 to XL).

## Conflict of interest

The authors declare that the research was conducted in the absence of any commercial or financial relationships that could be construed as a potential conflict of interest.

## Publisher's note

All claims expressed in this article are solely those of the authors and do not necessarily represent those of their affiliated organizations, or those of the publisher, the editors and the reviewers. Any product that may be evaluated in this article, or claim that may be made by its manufacturer, is not guaranteed or endorsed by the publisher.

## Abbreviations

WMH: white matter hyperintensity
EPVS: enlarged perivascular spaces
CMBs: cerebral microbleeds
CSVD: cerebral small vessel disease
MRI: magnetic resonance imaging
HRV: heart rate variability
AHR: average heart rate
ANS: autonomic nervous system
SDNN: the standard deviation of normal-to-normal intervals.

## References

[1] Pantoni L. Cerebral small vessel disease: from pathogenesis and clinical characteristics to therapeutic challenges. Lancet Neurol. (2010) 9:689–701. 10.1016/S1474-4422(10)70104-6 20610345

[2] Wardlaw J Smith C Dichgans M. Small vessel disease: mechanisms and clinical implications. Lancet Neurol. (2019) 18:684–96. 10.1016/S1474-4422(19)30079-1 31097385

[3] Wardlaw J Smith C Dichgans M. Mechanisms of sporadic cerebral small vessel disease: insights from neuroimaging. Lancet Neurol. (2013) 12:483–97. 10.1016/S1474-4422(13)70060-7 23602162 PMC3836247

[4] Jackson C Sudlow C. Are lacunar strokes really different? A systematic review of differences in risk factor profiles between lacunar and nonlacunar infarcts. Stroke. (2005) 36:891–901. 10.1161/01.STR.0000157949.34986.30 15761206 PMC2577185

[5] Yamaguchi Y Wada M Sato H Nagasawa H Koyama S Takahashi Y. Impact of ambulatory blood pressure variability on cerebral small vessel disease progression and cognitive decline in community-based elderly Japanese. Am J Hypertens. (2014) 27:1257–67. 10.1093/ajh/hpu045 24651635

[6] Chen Y Ni Z Li W Xiao W Liu Y Liang W. Diurnal blood pressure and heart rate variability in hypertensive patients with cerebral small vessel disease: a case-control study. J Stroke Cerebrovasc Dis. (2021) 30:105673. 10.1016/j.jstrokecerebrovasdis.2021.105673 33631472

[7] Dekker J Crow R Folsom A Hannan P Liao D Swenne C. Low heart rate variability in a 2-minute rhythm strip predicts risk of coronary heart disease and mortality from several causes: the ARIC Study. Atherosclerosis risk in communities. Circulation. (2000) 102:1239–44. 10.1161/01.CIR.102.11.1239 10982537

[8] Fyfe-Johnson A Muller C Alonso A Folsom A Gottesman R Rosamond W. Heart rate variability and incident stroke: the atherosclerosis risk in communities study. Stroke. (2016) 47:1452–8. 10.1161/STROKEAHA.116.012662 27217501 PMC4880420

[9] Guan L Wang Y Claydon VE Mazowita G Wang Y Brant R. Autonomic parameter and stress profile predict secondary ischemic events after transient ischemic attack or minor stroke. Stroke. (2019) 50:2007–15. 10.1161/STROKEAHA.118.022844 31238826

[10] Zhang R Zuckerman JH Iwasaki K Wilson TE Crandall CG Levine BD. Autonomic neural control of dynamic cerebral autoregulation in humans. Circulation. (2002) 106:1814–20. 10.1161/01.CIR.0000031798.07790.FE 12356635

[11] Hamner JW Tan CO Lee K Cohen MA Taylor JA. Sympathetic control of the cerebral vasculature in humans. Stroke. (2010) 41:102–9. 10.1161/STROKEAHA.109.557132 20007920 PMC2814242

[12] Yamaguchi Y Wada M Sato H Nagasawa H Koyama S Takahashi Y. Impact of nocturnal heart rate variability on cerebral small-vessel disease progression: a longitudinal study in community-dwelling elderly Japanese. Hypertens Res. (2015) 38:564–9. 10.1038/hr.2015.38 25787037

[13] Del Brutto OH Mera RM Costa AF Castillo PR. Effect of heart rate variability on the association between the apnea-hypopnea index and cerebral small vessel disease. Stroke. (2019) 50:2486–91. 10.1161/STROKEAHA.119.026095 31345136

[14] Liao DP Carnethon M Evans GW Cascio WE Heiss G. Lower heart rate variability is associated with the development of coronary heart disease in individuals with diabetes - The Atherosclerosis Risk in Communities (ARIC) Study. Diabetes. (2002) 51:3524–31. 10.2337/diabetes.51.12.3524 12453910

[15] Wardlaw JM Smith EE Biessels GJ Cordonnier C Fazekas F Frayne R. Neuroimaging standards for research into small vessel disease and its contribution to ageing and neurodegeneration. Lancet Neurol. (2013) 12:822–38. 10.1016/S1474-4422(13)70124-8 23867200 PMC3714437

[16] Fazekas F Schmidt R Scheltens P. Pathophysiologic mechanisms in the development of age-related white matter changes of the brain. Dement Geriatr Cogn. (1998) 9:2–5. 10.1159/000051182 9716237

[17] Smith EE Saposnik G Biessels GJ Doubal FN Fornage M Gorelick PB. Prevention of stroke in patients with silent cerebrovascular disease: a scientific statement for healthcare professionals from the American Heart Association/American Stroke Association. Stroke. (2017) 48:E44–71. 10.1161/STR.0000000000000116 27980126

[18] Zhu YC Tzourio C Soumare A Mazoyer B Dufouil C Chabriat H. Severity of dilated virchow-robin spaces is associated with age, blood pressure, and MRI markers of small vessel disease a population-based study. Stroke. (2010) 41:2483–90. 10.1161/STROKEAHA.110.591586 20864661

[19] Nakanishi K Jin ZZ Homma S Elkind MSV Rundek T Lee SC. Association between heart rate and subclinical cerebrovascular disease in the elderly. Stroke. (2018) 49:319–24. 10.1161/STROKEAHA.117.019355 29284731 PMC5870891

[20] Yano Y. Nocturnal heart rate and cerebrovascular disease. Hypertens Res. (2015) 38:528–9. 10.1038/hr.2015.58 25876829

[21] Assante R Acampa W Zampella E Arumugam P Nappi C Gaudieri V. Coronary atherosclerotic burden vs. coronary vascular function in diabetic and nondiabetic patients with normal myocardial perfusion: a propensity score analysis. Eur J Nucl Med Mol I. (2017) 44:1129–35. 10.1007/s00259-017-3671-y 28293706

[22] Robles-Cabrera A Torres-Arellano JM Fossion R Lerma C. Dependence of heart rate variability indices on the mean heart rate in women with well-controlled type 2 diabetes. J Clin Med. (2021) 10:386. 10.3390/jcm10194386 34640404 PMC8509544

[23] Kamble PG Theorell-Haglow J Wiklund U Franklin KA Hammar U Lindberg E. Sleep apnea in men is associated with altered lipid metabolism, glucose tolerance, insulin sensitivity, and body fat percentage. Endocrine. (2020) 70:48–57. 10.1007/s12020-020-02369-3 32562183 PMC7524823

[24] Chistiakov DA Ashwell KW Orekhov AN Bobryshev YV. Innervation of the arterial wall and its modification in atherosclerosis. Auton Neurosci-Basic. (2015) 193:7–11. 10.1016/j.autneu.2015.06.005 26164815

[25] Koichubekov BK Sorokina MA Laryushina YM Turgunova LG Korshukov IV. Nonlinear analyses of heart rate variability in hypertension. Annales de cardiologie et d'angeiologie. (2018) 67:174–9. 10.1016/j.ancard.2018.04.014 29753421

[26] Zhao MX Guan L Wang YL. The association of autonomic nervous system function with ischemic stroke, and treatment strategies. Front Neurol. (2020) 10:e01411. 10.3389/fneur.2019.01411 32038467 PMC6987371

[27] Tegegne BS Man TF van Roon AM Riese H Snieder H. Determinants of heart rate variability in the general population: the lifelines cohort study. Heart Rhythm. (2018) 15:1552–8. 10.1016/j.hrthm.2018.05.006 29753022

[28] Niu SW Huang JC Chen SC Lin HYH Kuo IC Wu PY. Association between age and changes in heart rate variability after hemodialysis in patients with diabetes. Front Aging Neurosci. (2018) 10:e00043. 10.3389/fnagi.2018.00043 29515436 PMC5826193

