# Supplementary material for: Heart rate variability is associated with cerebral small vessel disease in patients with diabetes
Source: Front Neurol. 2022 Nov 10;13:989064. doi: 10.3389/fneur.2022.989064 (PMC9685533; doi:10.3389/fneur.2022.989064)
Supplement: Supplementary file 1 [file Data_Sheet_1.docx]

**Supplementary materials**

**Table S1. Parameters for conventional MRI sequences.**

| **Sequences** | **T1WI** | **T2WI** | **FLAIR** | **DWI** | **SWI** |
| --- | --- | --- | --- | --- | --- |
| **TR, ms** | 2390 | 5200 | 7902 | 5400 | 30 |
| **TE, ms** | 10.69 | 107.41 | 140.45 | 90.30 | 20 |
| **FA, °** | 111 | 142 | 150 | 90 | 15 |
| **Slice thickness, mm** | 5 | 5 | 5 | 5 | 2 |
| **FOV, mm^2^** | 240 × 230 | 240 × 230 | 240× 230 | 230 × 230 | 230×230 |

Abbreviations: T1WI = T1-weighted imaging; T2WI = T2-weighted imaging; FLAIR = fluid-attenuated inversion recovery; DWI = diffusion-weighted imaging; TR = repetition time; TE = echo time; FA = flip angle; FOV = field of view.

**Table S2 Characteristics of the total study population according to total burden of CSVD**

|  | **CSVD 0**  **n = 47** | **CSVD 1**  **n = 85** | **CSVD 2**  **n =112** | **CSVD 3**  **n =123** | **CSVD 4**  **n = 69** | **P-value** |
| --- | --- | --- | --- | --- | --- | --- |
| **Age, years, median (IQR)** | 53.0 (50.0–55.0) | 57.0 (55.0–61.0) | 63.0 (59.0–65.8) | 65.0 (70.0–74.0) | 73.0 (68.3–78.8) | < 0.001 |
| **Male sex, n (%)** | 21(44.7) | 40 (47.1) | 56 (50.0) | 63 (51.2) | 35 (51.5) | 0.924 |
| **Smoking, n (%)** | 11 (23.9) | 30 (35.3) | 41 (36.6) | 45 (36.6) | 30 (44.1) | 0.265 |
| **Alcohol use, n (%)** | 8 (17.4) | 23 (27.1) | 34 (30.4） | 30 (24.4) | 19 (27.9) | 0.494 |
| **Hypertension, n (%)** | 22 (46.8) | 50 (58.8) | 77 (68.8) | 96 (78.0) | 64 (94.1) | < 0.001 |
| **Diabetes, n (%)** | 11 (23.4) | 18 (21.2) | 29 (25.7) | 42 (34.1) | 23 (33.8) | 0.171 |
| **Hyperlipidemia, n (%)** | 23 (48.9) | 33 (38.8) | 40 (35.7) | 40 (32.5) | 24 (35.3) | 0.377 |
| **Using of antihypertensive**  **drugs, n (%)** | 16 (34.0) | 35 (41.2) | 53 (47.3) | 72 (58.5) | 43 (63.2) | 0.003 |
| **Using of statin drugs, n(%)** | 16 (34.0) | 23 (27.1) | 25 (22.3) | 25 (20.3) | 14 (20.6) | 0.334 |
| **SBP, mm Hg, median (IQR)** | 131.0 (124.0–142.0) | 132.0 (123.0–141.5) | 131.5 (124.0–143.8) | 134.0 (127.0–144.0) | 138.0 (127.3–147.8) | 0.249 |
| **DBP, mm Hg, median (IQR)** | 79.0 (74.0–87.0) | 79.0 (73.5–85.0) | 78.5 (72.3–85.8) | 79.0 (73.0–84.0) | 77.0 (71.0–83.8) | 0.579 |
| **Glucose, mmol/L, median (IQR)** | 5.8 (5.5–6.2) | 5.8 (5.5–6.1) | 5.8 (5.6–6.3) | 5.8 (5.6–6.3) | 5.8 (5.7–6.3) | 0.290 |
| **HbA1c level, %, median (IQR)** | 5.2 (4.8–5.9) | 5.1 (4.7–5.6) | 5.1 (4.7–5.8) | 5.1 (4.8–5.6) | 5.4 (5.0–6.0) | 0.068 |
| **TC, mmol/L, median (IQR)** | 4.0 (3.4–4.7) | 3.7 (3.1–4.4) | 4.0 (3.2–4.7) | 3.8 (3.1–4.4) | 3.7 (3.1–4.4) | 0.475 |
| **TG, mmol/L, median (IQR)** | 1.4 (1.0–1.9) | 1.2 (0.9–1.9) | 1.2 (0.9–1.6) | 1.1 (0.8–1.6) | 1.1 (0.9–1.7) | 0.723 |
| **HDL, mmol/L, median (IQR)** | 1.1 (0.9–1.3) | 1.1 (0.9–1.3) | 1.1 (0.9–1.3) | 1.1 (0.9–1.3) | 1.0 (0.9–1.2) | 0.718 |
| **LDL, mmol/L,median (IQR)** | 2.4 (1.8–3.1) | 2.2 (1.5–2.8) | 2.6 (1.9–3.1) | 2.3 (1.7–2.7) | 2.3 (1.7–2.9) | 0.123 |
| **CSVD markers** |  |  |  |  |  |  |
| **Presence of severe WMH, n (%)** | – | 30 (35.3) | 79 (70.5) | 111 (90.2) | 69 (100.0) | < 0.001 |
| **Presence of Lacunes, n (%)** | – | 25 (29.4) | 55 (49.1) | 108 (87.8) | 69 (100.0) | < 0.001 |
| **Presence of EPVS, n (%)** | – | 3 (3.5) | 68 (60.7) | 110 (89.4) | 69 (100.0) | < 0.001 |
| **Presence of CMBs, n (%)** | – | 27 (31.8) | 22 (19.6) | 40 (32.5) | 69 (100.0) | < 0.001 |

Abbreviations: SBP = systolic blood pressure; DBP = diastolic blood pressure; TC = total cholesterol; TG = triglyceride; HDL = high-density lipoprotein; LDL = low-density lipoprotein; HAb1c = hemoglobin A1c; WMH = white matter hyperintensity; EPVS = enlarged perivascular spaces; CMBs = cerebral microbleeds.

**Table S3 Characteristics of the diabetic individuals according to total burden of CSVD**

|  | **CSVD 0**  **n = 11** | **CSVD 1**  **n = 18** | **CSVD 2**  **n =28** | **CSVD 3**  **n =42** | **CSVD 4**  **n = 23** | **P-value** |
| --- | --- | --- | --- | --- | --- | --- |
| **Age, years, median (IQR)** | 54.0 (51.0–56.0) | 60.5 (56.0–65.0) | 62.0 (53.3–66.0) | 68.0 (62.8–73.0) | 74.0 (70.0–78.0) | < 0.001 |
| **Male sex, n (%)** | 7 (63.6) | 11 (61.1) | 18 (64.3) | 20 (47.6) | 14 (60.9) | 0.631 |
| **Smoking, n (%)** | 4 (36.4) | 9 (50.0) | 14 (50.0) | 17 (40.5) | 14 (60.9) | 0.534 |
| **Alcohol use, n (%)** | 3 (27.4) | 8 (44.4) | 13 (46.4） | 11 (26.2) | 7 (30.4) | 0.372 |
| **Hypertension, n (%)** | 7 (63.6) | 13 (72.2) | 22 (78.6) | 38 (90.5) | 23 (100.0) | < 0.019 |
| **Hyperlipidemia, n (%)** | 7 (63.6) | 7 (38.9) | 7 (25.0) | 17 (40.5) | 12 (52.2) | 0.164 |
| **Using of antihypertensive**  **drugs, n (%)** | 6 (54.5) | 10 (55.6) | 12 (42.9) | 26 (61.9) | 15 (65.2) | 0.500 |
| **Using of statin drugs, n(%)** | 6 (54.5) | 6 (33.3) | 6 (21.4) | 12 (28.6) | 9 (39.1) | 0.310 |
| **SBP, mm Hg, median (IQR)** | 140.0 (123.0–149.0) | 133.5 (129.3–142.0) | 140.5. (125.8–156.5) | 139.0 (129.0–146.3) | 141.0 (131.0–152.0) | 0.758 |
| **DBP, mm Hg, median (IQR)** | 81.0 (78.0–94.0) | 78.0 (72.0–82.0) | 78.5 (72.5–87.5) | 79.0 (73.0–84.0) | 74.0 (70.0–82.0) | 0.155 |
| **Glucose, mmol/L, median (IQR)** | 7.2 (6.8–7.4) | 6.8 (6.4–7.9) | 6.9 (6.5–8.3) | 6.6 (6.3–7.3) | 6.5 (6.1–8.4) | 0.324 |
| **HbA1c level, %, median (IQR)** | 6.7 (5.7–7.7) | 6.0 (5.1–7.5) | 7.0 (5.3–9.4) | 5.7 (5.2–6.9) | 6.8 (5.3–9.5) | 0.076 |
| **TC, mmol/L, median (IQR)** | 4.0 (3.2–4.7) | 3.2 (2.6–3.8) | 3.8 (2.7–4.5) | 3.6 (2.9–4.2) | 4.1(3.4–5.0) | 0.100 |
| **TG, mmol/L, median (IQR)** | 1.9 (1.4–2.3) | 1.1 (1.0–1.8) | 1.3 (1.1–1.6) | 1.3 (0.9–1.8) | 1.5 (0.9–1.9) | 0.243 |
| **HDL, mmol/L, median (IQR)** | 1.0 (0.8–1.2) | 1.0 (0.8–1.2) | 0.9 (0.9–1.1) | 1.0 (0.9–1.2) | 1.0 (0.9–1.2) | 0.956 |
| **LDL, mmol/L,median (IQR)** | 2.4 (1.7–2.9) | 1.7 (1.3–2.3) | 2.4 (1.4–2.8) | 2.1 (1.4–2.7) | 2.5 (1.8–3.3) | 0.066 |
| **CSVD markers** |  |  |  |  |  |  |
| **Presence of severe WMH, n (%)** | – | 4 (22.2) | 19 (67.9) | 32 (76.2) | 23 (100.0) | < 0.001 |
| **Presence of Lacunes, n (%)** | – | 5 (27.8) | 14 (50.0) | 37 (88.1) | 23 (100.0) | < 0.001 |
| **Presence of EPVS, n (%)** | – | 8 (44.4) | 21 (75.0) | 38 (90.5) | 23 (100.0) | < 0.001 |
| **Presence of CMBs, n (%)** | – | 1 (5.6) | 2 (7.1) | 19 (45.2) | 23 (100.0) | < 0.001 |

Abbreviations: SBP = systolic blood pressure; DBP = diastolic blood pressure; TC = total cholesterol; TG = triglyceride; HDL = high-density lipoprotein; LDL = low-density lipoprotein; HAb1c = hemoglobin A1c; WMH = white matter hyperintensity; EPVS = enlarged perivascular spaces; CMBs = cerebral microbleeds.

**Table S4 Characteristics of the nondiabetic individuals according to total burden of CSVD**

|  | **CSVD 0**  **n = 36** | **CSVD 1**  **n = 67** | **CSVD 2**  **n =84** | **CSVD 3**  **n =81** | **CSVD 4**  **n = 45** | **P-value** |
| --- | --- | --- | --- | --- | --- | --- |
| **Age, years, median (IQR)** | 52.0 (50.0–55.0) | 57.0 (54.0–60.0) | 63.0 (60.0–65.0) | 70.0 (66.0–75.5) | 72.0 (68.0–80.5) | < 0.001 |
| **Male sex, n (%)** | 14 (38.9) | 29 (43.3) | 38 (45.2) | 43 (53.1) | 21 (46.7) | 0.630 |
| **Smoking, n (%)** | 7 (19.4) | 21 (31.3) | 27 (32.1) | 28 (34.6) | 16 (35.6) | 0.537 |
| **Alcohol use, n (%)** | 5 (13.9) | 15 (22.4) | 21 (25.0） | 19 (23.5) | 12 (26.7) | 0.691 |
| **Hypertension, n (%)** | 15 (41.7) | 37 (55.2) | 55 (65.5) | 58 (71.6) | 41 (91.1) | < 0.001 |
| **Hyperlipidemia, n (%)** | 16 (44.4) | 26 (38.8) | 33 (39.3) | 23 (28.4) | 12 (26.7) | 0.246 |
| **Using of antihypertensive**  **drugs, n (%)** | 10 (27.8) | 25 (37.3) | 41 (48.8) | 46 (56.8) | 28 (62.2) | 0.004 |
| **Using of statin drugs, n(%)** | 10 (27.8) | 17 (25.4) | 19 (22.6) | 13 (16.0) | 5 (11.1) | 0.214 |
| **SBP, mm Hg, median (IQR)** | 131.0 (124.0–140.0) | 132.0 (123.0–141.0) | 131.0 (123.0–140.0) | 132.0 (125.0–141.0) | 136.0 (124.0–144.0) | 0.607 |
| **DBP, mm Hg, median (IQR)** | 78.0 (72.3–84.8) | 79.0 (74.0–85.0) | 78.5 (72.3–85.0) | 79.0 (23.0–84.0) | 78.0 (72.0–85.0) | 0.872 |
| **Glucose, mmol/L, median (IQR)** | 5.6 (5.3–6.0) | 5.7 (5.5–5.9) | 5.7 (5.5–5.9) | 5.6 (5.5–5.8) | 5.7 (5.5–5.9) | 0.621 |
| **HbA1c level, %, median (IQR)** | 5.0 (4.7–5.4) | 5.0 (4.6–5.4) | 4.9 (4.7–5.3) | 5.0 (4.8–5.3) | 5.2 (4.9–5.6) | 0.114 |
| **TC, mmol/L, median (IQR)** | 4.1 (3.4–4.7) | 3.8 (3.2–4.4) | 4.1 (3.3–4.7) | 4.0 (3.1–4.6) | 3.6 (3.0–4.3) | 0.259 |
| **TG, mmol/L, median (IQR)** | 1.3 (1.0–1.8) | 1.2 (0.9–1.9) | 1.1 (0.8–1.6) | 1.1 (0.8–1.4) | 1.1 (0.8–1.5) | 0.377 |
| **HDL, mmol/L, median (IQR)** | 1.1 (1.0–1.3) | 1.1 (0.9–1.3) | 1.1 (0.9–1.3) | 1.1 (0.9–1.3) | 1.0 (0.9–1.2) | 0.679 |
| **LDL, mmol/L,median (IQR)** | 2.4 (2.1–3.2) | 2.4 (1.7–2.9) | 2.6 (2.0–3.1) | 2.3 (1.8–2.8) | 2.2 (1.6–2.7) | 0.073 |
| **CSVD markers** |  |  |  |  |  |  |
| **Presence of severe WMH, n (%)** | – | 26 (38.8) | 60 (71.4) | 79 (97.5) | 45 (100.0) | < 0.001 |
| **Presence of Lacunes, n (%)** | – | 20 (29.9) | 41 (48.8) | 71 (87.7) | 45 (100.0) | < 0.001 |
| **Presence of EPVS, n (%)** | – | 19 (28.4) | 47 (56.0) | 72 (88.9) | 45 (100.0) | < 0.001 |
| **Presence of CMBs, n (%)** | – | 2 (3.0) | 20 (23.8) | 21 (25.9) | 45 (100.0) | < 0.001 |

1. Pantoni, L., Cerebral small vessel disease: from pathogenesis and clinical characteristics to therapeutic challenges. The Lancet. Neurology, 2010. 9(7): p. 689-701.

2. Wardlaw, J., C. Smith, and M. Dichgans, Small vessel disease: mechanisms and clinical implications. The Lancet. Neurology, 2019. 18(7): p. 684-696.

3. Wardlaw, J., C. Smith, and M. Dichgans, Mechanisms of sporadic cerebral small vessel disease: insights from neuroimaging. The Lancet. Neurology, 2013. 12(5): p. 483-97.

4. Yamaguchi, Y., et al., Impact of ambulatory blood pressure variability on cerebral small vessel disease progression and cognitive decline in community-based elderly Japanese. American journal of hypertension, 2014. 27(10): p. 1257-67.

5. Chen, Y., et al., Diurnal Blood Pressure and Heart Rate Variability in Hypertensive Patients with Cerebral Small Vessel Disease: A Case-Control Study. Journal of stroke and cerebrovascular diseases : the official journal of National Stroke Association, 2021. 30(5): p. 105673.

6. Dekker, J., et al., Low heart rate variability in a 2-minute rhythm strip predicts risk of coronary heart disease and mortality from several causes: the ARIC Study. Atherosclerosis Risk In Communities. Circulation, 2000. 102(11): p. 1239-44.

7. Fyfe-Johnson, A., et al., Heart Rate Variability and Incident Stroke: The Atherosclerosis Risk in Communities Study. Stroke, 2016. 47(6): p. 1452-8.

8. Guan, L., et al., Autonomic Parameter and Stress Profile Predict Secondary Ischemic Events After Transient Ischemic Attack or Minor Stroke. Stroke, 2019. 50(8): p. 2007-2015.

9. Yamaguchi, Y., et al., Impact of nocturnal heart rate variability on cerebral small-vessel disease progression: a longitudinal study in community-dwelling elderly Japanese. Hypertension research : official journal of the Japanese Society of Hypertension, 2015. 38(8): p. 564-9.

10. Del Brutto, O., et al., Effect of Heart Rate Variability on the Association Between the Apnea-Hypopnea Index and Cerebral Small Vessel Disease. Stroke, 2019. 50(9): p. 2486-2491.

11. van Sloten, T., et al., Cerebral microvascular complications of type 2 diabetes: stroke, cognitive dysfunction, and depression. The lancet. Diabetes & endocrinology, 2020. 8(4): p. 325-336.

12. Liao, D., et al., Lower heart rate variability is associated with the development of coronary heart disease in individuals with diabetes: the atherosclerosis risk in communities (ARIC) study. Diabetes, 2002. 51(12): p. 3524-31.

13. Fazekas, F., R. Schmidt, and P. Scheltens, Pathophysiologic mechanisms in the development of age-related white matter changes of the brain. Dementia and geriatric cognitive disorders, 1998: p. 2-5.

14. Fazekas, F., R. Schmidt, and P. Scheltens, Pathophysiologic mechanisms in the development of age-related white matter changes of the brain. Dementia and geriatric cognitive disorders, 1998: p. 2-5.

16. Ergun, U., et al., Power spectral analysis of heart rate variability: normal values of subjects over 60 years old. The International journal of neuroscience, 2008. 118(8): p. 1165-73.

17. Nakanishi, K., et al., Association Between Heart Rate and Subclinical Cerebrovascular Disease in the Elderly. Stroke, 2018. 49(2): p. 319-324.

18. Yano, Y., Nocturnal heart rate and cerebrovascular disease. Hypertension research : official journal of the Japanese Society of Hypertension, 2015. 38(8): p. 528-9.

19. Robles-Cabrera, A., et al., Dependence of Heart Rate Variability Indices on the Mean Heart Rate in Women with Well-Controlled Type 2 Diabetes. Journal of clinical medicine, 2021. 10(19).

20. Kamble, P., et al., Sleep apnea in men is associated with altered lipid metabolism, glucose tolerance, insulin sensitivity, and body fat percentage. Endocrine, 2020. 70(1): p. 48-57.

21. Zhang, R., et al., Autonomic neural control of dynamic cerebral autoregulation in humans. Circulation, 2002. 106(14): p. 1814-20.

22. Hamner, J., et al., Sympathetic control of the cerebral vasculature in humans. Stroke, 2010. 41(1): p. 102-9.

23. Chistiakov, D., et al., Innervation of the arterial wall and its modification in atherosclerosis. Autonomic neuroscience : basic & clinical, 2015. 193: p. 7-11.

24. Koichubekov, B., et al., Nonlinear analyses of heart rate variability in hypertension. Annales de cardiologie et d'angeiologie, 2018. 67(3): p. 174-179.

25. Zhao, M., L. Guan, and Y. Wang, The Association of Autonomic Nervous System Function With Ischemic Stroke, and Treatment Strategies. Frontiers in neurology, 2019. 10: p. 1411.

26. Tegegne, B., et al., Determinants of heart rate variability in the general population: The Lifelines Cohort Study. Heart rhythm, 2018. 15(10): p. 1552-1558.

27. Niu, S., et al., Association between Age and Changes in Heart Rate Variability after Hemodialysis in Patients with Diabetes. Frontiers in aging neuroscience, 2018. 10: p. 43.

28. Wardlaw, J., et al., Neuroimaging standards for research into small vessel disease and its contribution to ageing and neurodegeneration. The Lancet. Neurology, 2013. 12(8): p. 822-38.
